# Large-scale contamination of microbial isolate genomes by Illumina PhiX control

**DOI:** 10.1186/1944-3277-10-18

**Published:** 2015-03-30

**Authors:** Supratim Mukherjee, Marcel Huntemann, Natalia Ivanova, Nikos C Kyrpides, Amrita Pati

**Affiliations:** 1 DOE Joint Genome Institute, Walnut Creek, CA, USA; 2 King Abdulaziz University, Jeddah, Saudi Arabia

**Keywords:** Next-generation sequencing, PhiX, Contamination, Comparative genomics

## Abstract

With the rapid growth and development of sequencing technologies, genomes have become the new go-to for exploring solutions to some of the world’s biggest challenges such as searching for alternative energy sources and exploration of genomic dark matter. However, progress in sequencing has been accompanied by its share of errors that can occur during template or library preparation, sequencing, imaging or data analysis. In this study we screened over 18,000 publicly available microbial isolate genome sequences in the Integrated Microbial Genomes database and identified more than 1000 genomes that are contaminated with PhiX, a control frequently used during Illumina sequencing runs. Approximately 10% of these genomes have been published in literature and 129 contaminated genomes were sequenced under the Human Microbiome Project. Raw sequence reads are prone to contamination from various sources and are usually eliminated during downstream quality control steps. Detection of PhiX contaminated genomes indicates a lapse in either the application or effectiveness of proper quality control measures. The presence of PhiX contamination in several publicly available isolate genomes can result in additional errors when such data are used in comparative genomics analyses. Such contamination of public databases have far-reaching consequences in the form of erroneous data interpretation and analyses, and necessitates better measures to proofread raw sequences before releasing them to the broader scientific community.

## Background

The ability to produce large numbers of high-quality, low-cost reads has revolutionized the field of microbiology [1-3]. Starting from a meager 1575 registered projects in September 2005, there has been a steady increase in the number of sequencing projects according to the Genomes OnLine Database [4]. As of November 17th 2014, there were 41,553 bacterial and archaeal isolate genome sequencing projects reported in GOLD [4,5]. This explosion of genome sequencing projects especially during the last 5 years has been largely catalyzed by the development of several next-generation sequencing platforms offering rapid and accurate genome information at a low cost. Among the different NGS technologies available commercially, the sequencing by synthesis technology [6] championed by Illumina [7] is the most widely used.

Despite its high accuracy, the Illumina sequencing platform does come with its share of challenges [8] that need to be addressed by the users of this technology. One such challenge is the protocol in which PhiX is used as a quality and calibration control for sequencing runs. PhiX is an icosahedral, nontailed bacteriophage with a single-stranded DNA. It has a tiny genome with 5386 nucleotides and was the first DNA genome to be sequenced by Fred Sanger [9]. Due to its small, well-defined genome sequence, PhiX has been commonly used as a control for Illumina sequencing runs. For the majority of its library preparations Illumina recommends using PhiX at a low concentration of 1%, which can be raised up to 40% for low diversity samples. Depending on the concentration of PhiX used, it can be spiked in the same lane along with the sample or used as a separate lane. Addition of PhiX as a sequencing control necessitates subsequent quality control steps to remove the sequences such that they do not get integrated as part of the target genome.

Here, we identify and catalog more than 1000 genomes in public databases (i.e. Genbank) that are contaminated with PhiX sequences and the approximately 10% of the genomes that are published in literature. In an era where sequencing data is growing exponentially along with the need to rapidly churn out novel sequences, our report serves as a reminder that it is equally important to develop effective downstream screening and quality control measures to prevent large-scale contamination of public databases. Since preliminary analyses of initial draft sequences lead to formulation of key scientific questions, contamination can result in misinterpretation of data and drawing of erroneous biological conclusions.

## Methods

We screened the current list of isolate microbial genomes in the Integrated Microbial Genomes (IMG v 4.0) [10] against the PhiX genome. The nucleotide sequence of each query genome was compared against PhiX using NCBI-BLASTn [11] and hits above a percent identity of 90% and e-value of 0.01 were retained. A hit was flagged as being contaminated with PhiX sequences if its total length was at least 80% of the length of the contig.

## Results

Among the isolate bacterial and archaeal genomes in IMG v4.0, 1230 scaffolds from 1041 genomes were contaminated with PhiX sequences, with 105 contaminated genomes published in literature (Additional files 1 and 2). A summary of the affected genomes, sequencing information and their sequence assembly method is displayed in Additional file 3. Sequences of these genomes were incorporated into IMG from NCBI Reference Sequence Database. Majority of the contaminated scaffolds (1216 out of 1230) have a 100% PhiX contamination, 11 scaffolds have a 99% contamination, 4 scaffolds have a contamination rate between 94–98% while PhiX sequences contaminated 83% of 1 scaffold (Additional file 1). Sixty-two genomes have multiple scaffolds (between 2 and 10 scaffolds each) that are contaminated with PhiX sequences. While the average length of contamination in such a single scaffold varies between 406 bp and 1878 bp, the total contamination per genome adds up to 4055–4777 bp (Table 1). Approximately 94% (979) genomes have a single scaffold each, with an average length of 5587 bp that is contaminated with PhiX (Table 1).

**Table 1:** Summary of genomes and their corresponding scaffolds contaminated with PhiX sequences

**Number of Genomes**	**Number of contaminated scaffolds/genome**	**Average contaminated sequence length (bp)/ scaffold**	**Average contaminated sequence length (bp)/ genome**
2	10	406	4055
5	9	476	4282
6	8	502	4017
3	7	627	4389
46	2–6	1878	4777
979	1	5587	5587

**Figure 1:**
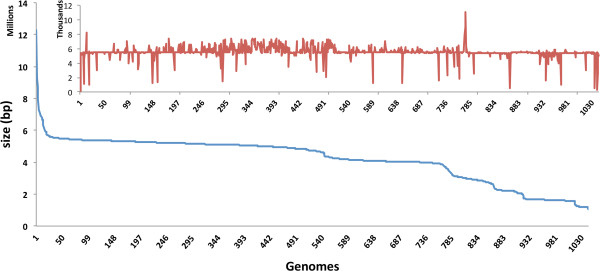
Genome size and contaminated sequence length (inset) of PhiX contaminated taxa.

The size of the genomes contaminated with PhiX varies from the tiny 1.05 Mb intracellular *Chlamydophila psittaci* *10_881_SC42 *[12-14] to the 12.2 Mb antifungal natural product synthesizing myxobacterium *Cystobacter fuscus* [15] (Figure 1). While the average length of contaminated sequence per genome is 5530 bp matching perfectly with the 5386 bp size of an entire PhiX genome, there is no direct correlation between the percentage of contamination and the size of the affected genome (Figure 1, inset). The source of contamination appears to be related to the sequencing center and its analysis and quality control pipeline. The PhiX contaminated genomes were sequenced by 54 different universities and sequencing centers; so it seems that the problem is quite widespread among sequencing groups (Additional file 3). Genomes from the Human Microbiome Project account for a little over 12% of the contaminated genomes (Additional file 3).

## Conclusions

The presence of PhiX sequences within individual genomes first attracted our attention while manually curating a small number of isolate genomes. Initially thought of as an exciting biological phenomenon or the result of horizontal gene transfer, after careful analyses, these scaffolds turned out to be nothing but sequencing artifacts. Sequencing centers generate massive amounts of data, which calls for strict quality control measures. The sheer volume of data being generated on a daily basis necessitates well-defined, automatic quality control protocols at source. Contaminated sequences once released to public databases typically trace thousands of analysis routes and can add to error propagation and incorrect hypotheses [16]. Thus, it is extremely important to detect contaminated sequences at the source and prevent them from affecting subsequent downstream analyses.

Contamination and sequence artifacts can come from multiple sources including but not limited to sequencing controls such as PhiX, cloning vectors, adapters, PCR primers, nucleic acid impurities present in reagents required for sample isolation and preparation and human error. Salter et al. [17] identified a wide range of contaminants from DNA extraction kits and other laboratory reagents affecting the outcome of culture-independent microbiota research; while Lusk [18] detected widespread contamination in four independent high throughput sequencing experiments. A study [19] scanning DNA sequences from The Thousand Genome Project [20] identified significant contamination by *Mycoplasma* sequences. While DNA contamination has been a long-standing issue in research laboratories, its potential long-term implications were highlighted recently in light of developments in high throughput sequencing and human microbiome research. A recent commentary published in Nature [21] summarizes the problem well.

Several tools have been developed over the years for quality control of raw sequence reads such as Phred [22], Sequence Scanner [23] (specifically for first generation sequence data) and NCBI’s VecScreen and UniVec [24,25] to get rid of contaminants of vector origin. More recent programs have been designed for analyzing NGS data such as TileQC [26], FastQC [27], PRINSEQ [28], NGS-QC [29], programs to detect contamination such as DeconSeq [30], as well as multi genome alignment (MGA) [31] and QC-Chain [32] which can provide both rapid QC and contamination filtering of NGS data. Such programs are meant to prevent release of contaminated sequences. However, our results from scanning publicly available microbial isolate genome sequences for contamination shows that large number of errors can be detected in spite of the easy availability of multiple quality control measures. The sheer volume of PhiX contaminated genomes is alarming and calls for implementation of stricter quality control measures especially at large genome centers with high rates of sequence turnover.

Detection of PhiX contamination encouraged us to expand our search further; we performed additional analysis looking for other sources of contamination and have identified genomes in public databases that are:

(a)  either a partial or complete mixtures of two or more strains

(b)  genomes contaminated with short fragments of two or more species

(c)  ‘isolate’ genomes where a complete genome is cloned inside another

The list of such genomes is available in Additional file 4 and their nucleotide sequences are available on a JGI public ftp site [33]. The IMG database has already implemented a quality control step to identify and remove these artifacts during data submission, and the sequence data in the system is free of PhiX contamination. We are currently in the process of cleaning up additional contaminated genomes. Most have already been removed from IMG completely or are being re-instated after cleaning up of contaminated scaffolds. At the same time, most of the PhiX contaminated genomes continue to exist in other public databases such as NCBI/RefSeq or Genbank and are easily accessible to researchers over the world. While we welcome the technological advances associated with NGS platforms and acknowledge their long-term benefits, we expect principal investigators (PI) of large-scale sequencing projects to be aware of the possible pitfalls and take corrective measures as necessary. For the genomes contaminated with PhiX sequences, we recommend individual PI’s to retract the corresponding sequences, remove contaminating scaffolds, and re-upload the clean sequences to public databases.

## Abbreviations

IMG: Integrated Microbial Genomes; HMP: Human Microbiome Project; GOLD: Genomes OnLine Database; NGS: next-generation sequencing; SBS: sequencing by synthesis.

## Competing interests

The authors declare that they have no competing interests.

## Authors’ contributions

AP and NI initiated the project. SM, AP and MH performed all analysis tasks. NI, NCK and AP performed validation of analysis. SM and AP wrote the paper. All authors read and approved the final manuscript.

## Supplementary Material

Additional file 1 Complete list of PhiX contaminated scaffolds, corresponding IMG Taxon IDs and their percentage of contamination. Click here for file

Additional file 2 List of genomes contaminated with PhiX that has been published in literature. Click here for file

Additional file 3 Detailed sequencing information of PhiX contaminated genomes. Click here for file

Additional file 4 List of non-PhiX contaminations that were detected and removed from the public IMG database. Click here for file
